# Reply: Functional similarity is more appropriate than functional redundancy

**DOI:** 10.1038/s44185-023-00030-6

**Published:** 2023-11-08

**Authors:** Nico Eisenhauer, Jes Hines, Matthias C. Rillig

**Affiliations:** 1 German Centre for Integrative Biodiversity Research (iDiv) Halle-Jena-Leipzig, Puschstr. 4, 04103 Leipzig, Germany; 2 Institute of Biology, Leipzig University, Puschstr. 4, 04103 Leipzig, Germany; 3 Freie Universität Berlin, Institute of Biology, Berlin, Germany; 4 Berlin-Brandenburg Institute of Advanced Biodiversity Research (BBIB), Berlin, Germany

**Keywords:** Ecology, Ecology

**replying to** Felícia M. Fischer and Francesco de Bello *npj Biodiversity* https://doi.org/10.1038/s44185-023-00029-z (2023)

**Fig. 1:**
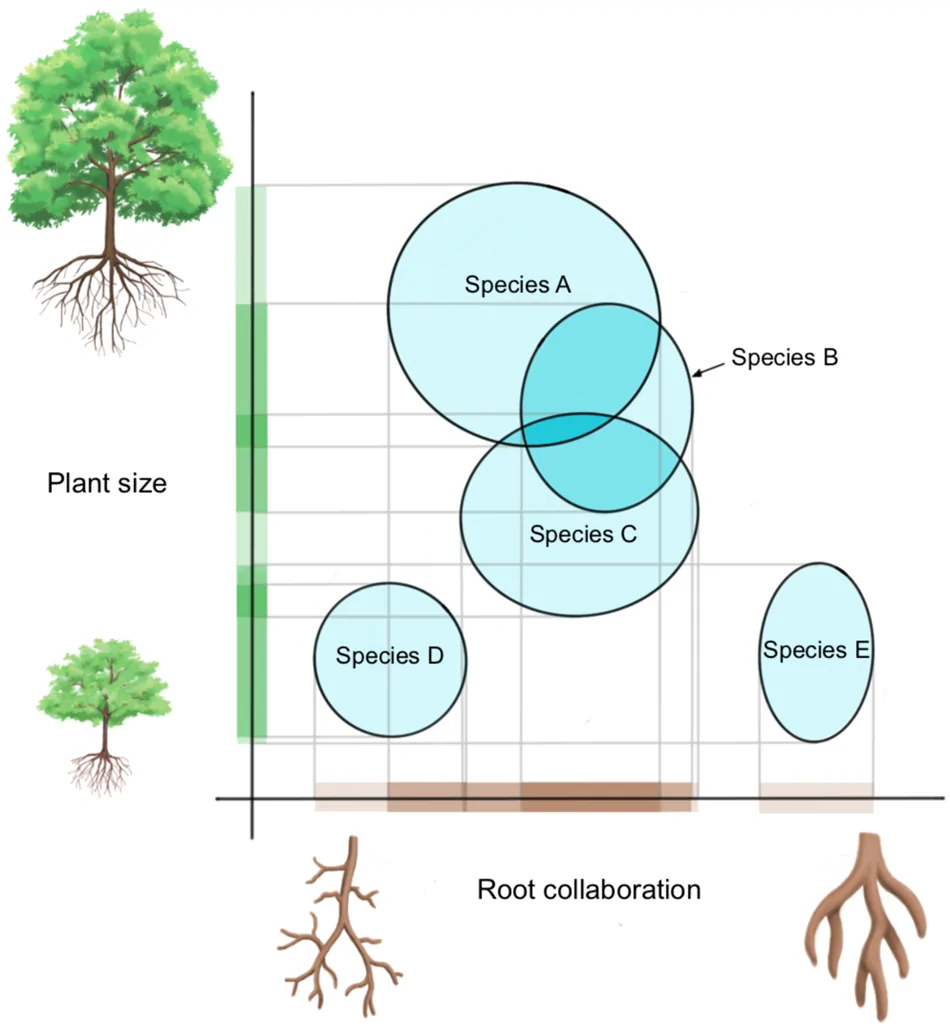
Conceptual figure displaying hypothetical species A–E in two-dimensional trait space. Species that strongly overlap with respect to one trait, such as plant size gradient (e.g., plant height or rooting depth), may be dissimilar with respect to a second trait, such as root collaboration gradient^14^. In this simple example, only two traits are displayed for simplicity, while ecological niches are highly multidimensional. Describing species as functionally similar is more appropriate than describing them as functionally redundant.

In our Comment, we outlined that the term functional redundancy (1) may have been overused from an ecological perspective and (2) can be dangerous and misleading in scientific communication^1^. As a constructive way forward, we proposed to use the concept of “functional similarity” with regard to specific ecosystem functions to better highlight the unique contributions of all coexisting species to ecosystem functioning (Fig. 1). Moreover, we argued that functional similarity better describes gradients in niche overlap while having a less negative connotation. We were motivated to propose this change in terminology because of the intense public discourse on biodiversity-related topics and the potential of misinterpretation that may be caused by the mostly negative connotation that is associated with redundancy (see common definitions below).

Fischer and de Bello (hereafter F23) suggest that the term “functional redundancy” has merits that should not be overlooked^2^. We appreciate several of their arguments, yet still question the utility of the term. The crux of their argument hinges upon appreciating a more nuanced interpretation of the term redundancy rather than abandoning it. In particular, they suggest that species may be redundant with respect to some traits (e.g., effect traits), but still differ with respect to other traits (e.g., response traits). We agree that species’ abiotic requirements and their role in ecosystem functioning are both important. Indeed, we recently described the role of environmental heterogeneity as a key component of species coexistence, biodiversity, and ecosystem functioning^3^. However, we maintain that it is problematic to describe species as functionally redundant when key aspects of their biology differ (Fig. 1). Nonetheless, we accept their call for added specificity and quantification of the detailed ways in which species are similar and how they differ. In this way, they emphasize the need to decompose species diversity, functional diversity, and functional redundancy into independent components. We agree this is a promising way forward^4,5^.

F23 argue that BEF experiments with random biodiversity loss scenarios may not reflect natural biodiversity loss that is highly trait dependent^2^. This basically repeats previous critiques^6^, but ignores the fact that environmental change is highly multifaceted and hard to predict^7,8^, and that recent comparisons between BEF experiments and real-world observations have provided consistent conclusions^9–11^.

F23 suggest that redundancy should be interpreted positively, and it should be perceived as reinforcing the safeness of ecosystems^2^. We appreciate the additional example, suggesting that redundancy has positive connotations in the field of engineering. Nonetheless, this merely emphasizes that this is a value-loaded term. In some cases, redundancy is perceived as “good” (e.g., it is associated with safety in engineering). In other cases, redundancy has a “bad” connotation (e.g., it is associated with being expendable in the workforce). The negative perception of being unnecessary is supported by the definition of “redundancy” in the *Cambridge Dictionary*: (1) a situation in which someone loses their job because their employer does not need them; (2) a situation in which something is unnecessary because it is more than is needed; (3) the unnecessary use of more than one word or phrase meaning the same thing^12^. Further synonyms include jobless, dismissed, sacked, unemployed, laid off, and out of work^12^.

Thus, redundancy in everyday language use, thus, clearly does not have a strong positive connotation. But, either way, we should be cognizant of misperceptions when using emotive language that may bias readers in unintended ways. Communicating about biodiversity is complex enough. Having to explain the intended meaning of the word “redundancy” places an unnecessary burden on that kind of communication and opens the door to misunderstandings that could be avoided by using a different term, as we propose. Importantly, much communication with the public is often done by science journalists and press offices, not by scientists who will be fully familiar with technical vocabulary.

“Similarity” and “dissimilarity” are concepts that are widely used in ecology (e.g., in community ecology) and also beyond. Although these terms also present some limitations^2^, their meaning is unlikely to give rise to misunderstanding, and they capture the essence of what they should express. We thus strongly suggest moving to this new terminology. Changing ecological terminology is commonly done^12,13^, but often awkward since different terms are used in parallel for a time, and thus any such change typically meets with resistance; but in the long run, replacing problematic terminology can come with tangible benefits for a field of science. As explained here and in our original paper, we think this is the case for the suggested replacement of “redundancy”^1^.
